# Supplementary material for: Modeling glioblastoma heterogeneity as a dynamic network of cell states
Source: Mol Syst Biol. 2021 Sep 16;17(9):e10105. doi: 10.15252/msb.202010105 (PMC8444284; doi:10.15252/msb.202010105)
Supplement: Supplementary file 6 — Source Data for Figure 5 [file MSB-17-e10105-s004.zip › Figure5A_sourcedata/GSEA_3017/nosigdb_stateE.GseaPreranked.1622795031093/DOROSZKO_U3180MG_CLUSTER_3.html]

Details for gene set DOROSZKO\_U3180MG\_CLUSTER\_3[GSEA]

|  || Dataset | state63017 |
| Phenotype | NoPhenotypeAvailable |
| Upregulated in class | na\_neg |
| GeneSet | DOROSZKO\_U3180MG\_CLUSTER\_3 |
| Enrichment Score (ES) | -0.6339735 |
| Normalized Enrichment Score (NES) | -3.7273846 |
| Nominal p-value | 0.0 |
| FDR q-value | 0.0 |
| FWER p-Value | 0.0 |
Table: GSEA Results Summary

  

Fig 1: Enrichment plot: DOROSZKO\_U3180MG\_CLUSTER\_3      
 Profile of the Running ES Score & Positions of GeneSet Members on the Rank Ordered List

  

| PROBE | GENE SYMBOL | GENE\_TITLE | RANK IN GENE LIST | RANK METRIC SCORE | RUNNING ES | CORE ENRICHMENT || 1 | PEG10 |  |  | 54 | 0.506 | -0.0775 | No |
| 2 | TCEAL9 |  |  | 137 | 0.343 | -0.1979 | No |
| 3 | CSRP2 |  |  | 247 | 0.264 | -0.3591 | No |
| 4 | DONSON |  |  | 274 | -0.251 | -0.3964 | No |
| 5 | SUPT16H |  |  | 276 | -0.251 | -0.3963 | No |
| 6 | MCMBP |  |  | 280 | -0.253 | -0.3992 | No |
| 7 | CHAC2 |  |  | 281 | -0.253 | -0.3976 | No |
| 8 | TUBA1C |  |  | 282 | -0.253 | -0.3959 | No |
| 9 | RMI2 |  |  | 292 | -0.257 | -0.4078 | No |
| 10 | H2AFY |  |  | 297 | -0.257 | -0.4121 | No |
| 11 | YEATS4 |  |  | 300 | -0.258 | -0.4135 | No |
| 12 | WDR34 |  |  | 303 | -0.258 | -0.4148 | No |
| 13 | HNRNPAB |  |  | 312 | -0.261 | -0.4251 | No |
| 14 | HMGA1 |  |  | 313 | -0.261 | -0.4234 | No |
| 15 | AHCY |  |  | 317 | -0.262 | -0.4262 | No |
| 16 | PSIP1 |  |  | 319 | -0.263 | -0.4261 | No |
| 17 | TUBG1 |  |  | 323 | -0.263 | -0.4289 | No |
| 18 | MAGOHB |  |  | 328 | -0.264 | -0.4332 | No |
| 19 | NCAPH2 |  |  | 331 | -0.264 | -0.4345 | No |
| 20 | RAD51C |  |  | 333 | -0.264 | -0.4343 | No |
| 21 | MCM5 |  |  | 337 | -0.265 | -0.4371 | No |
| 22 | HDAC2 |  |  | 348 | -0.268 | -0.4503 | No |
| 23 | NUDT15 |  |  | 356 | -0.270 | -0.4591 | No |
| 24 | POLE3 |  |  | 367 | -0.273 | -0.4723 | No |
| 25 | POP7 |  |  | 370 | -0.273 | -0.4735 | No |
| 26 | TMEM237 |  |  | 383 | -0.276 | -0.4897 | No |
| 27 | SLBP |  |  | 386 | -0.277 | -0.4909 | No |
| 28 | NFATC2IP |  |  | 397 | -0.283 | -0.5041 | No |
| 29 | DERA |  |  | 402 | -0.284 | -0.5082 | No |
| 30 | LSM4 |  |  | 406 | -0.284 | -0.5109 | No |
| 31 | PPIH |  |  | 407 | -0.284 | -0.5091 | No |
| 32 | SIVA1 |  |  | 412 | -0.286 | -0.5133 | No |
| 33 | CDK2 |  |  | 422 | -0.290 | -0.5249 | No |
| 34 | FANCG |  |  | 424 | -0.291 | -0.5245 | No |
| 35 | OIP5 |  |  | 426 | -0.291 | -0.5242 | No |
| 36 | MYBL1 |  |  | 432 | -0.294 | -0.5298 | No |
| 37 | LSM8 |  |  | 441 | -0.297 | -0.5398 | No |
| 38 | PPIF |  |  | 442 | -0.297 | -0.5379 | No |
| 39 | KPNB1 |  |  | 445 | -0.298 | -0.5390 | No |
| 40 | CDC7 |  |  | 451 | -0.299 | -0.5446 | No |
| 41 | ACYP1 |  |  | 454 | -0.301 | -0.5457 | No |
| 42 | CHTF18 |  |  | 455 | -0.301 | -0.5438 | No |
| 43 | HNRNPD |  |  | 456 | -0.301 | -0.5418 | No |
| 44 | MCM4 |  |  | 457 | -0.301 | -0.5399 | No |
| 45 | UBR7 |  |  | 459 | -0.302 | -0.5395 | No |
| 46 | DNMT1 |  |  | 466 | -0.304 | -0.5465 | No |
| 47 | SMC1A |  |  | 472 | -0.307 | -0.5520 | No |
| 48 | RECQL4 |  |  | 493 | -0.311 | -0.5800 | No |
| 49 | RFC2 |  |  | 498 | -0.313 | -0.5839 | No |
| 50 | PA2G4 |  |  | 501 | -0.314 | -0.5849 | No |
| 51 | GGH |  |  | 504 | -0.314 | -0.5859 | No |
| 52 | BARD1 |  |  | 510 | -0.318 | -0.5914 | No |
| 53 | HAUS1 |  |  | 518 | -0.320 | -0.5998 | No |
| 54 | POLA2 |  |  | 521 | -0.320 | -0.6008 | No |
| 55 | SUZ12 |  |  | 524 | -0.322 | -0.6017 | No |
| 56 | LSM3 |  |  | 530 | -0.323 | -0.6071 | No |
| 57 | CEP135 |  |  | 532 | -0.325 | -0.6065 | No |
| 58 | ACTL6A |  |  | 533 | -0.325 | -0.6045 | No |
| 59 | RFC5 |  |  | 534 | -0.326 | -0.6024 | No |
| 60 | GPN3 |  |  | 537 | -0.328 | -0.6033 | No |
| 61 | PRIM1 |  |  | 539 | -0.328 | -0.6027 | No |
| 62 | TPRKB |  |  | 540 | -0.329 | -0.6006 | No |
| 63 | CEP57 |  |  | 545 | -0.330 | -0.6045 | No |
| 64 | MSH2 |  |  | 548 | -0.332 | -0.6054 | No |
| 65 | MCM3 |  |  | 560 | -0.340 | -0.6196 | No |
| 66 | HAT1 |  |  | 561 | -0.340 | -0.6175 | No |
| 67 | RAD1 |  |  | 564 | -0.341 | -0.6183 | No |
| 68 | TCF19 |  |  | 569 | -0.342 | -0.6221 | No |
| 69 | NUDT5 |  |  | 572 | -0.345 | -0.6229 | No |
| 70 | CKLF |  |  | 574 | -0.347 | -0.6222 | No |
| 71 | CCDC15 |  |  | 576 | -0.347 | -0.6215 | No |
| 72 | HMGB3 |  |  | 577 | -0.348 | -0.6193 | No |
| 73 | CENPX |  |  | 578 | -0.348 | -0.6170 | No |
| 74 | RCC1 |  |  | 582 | -0.350 | -0.6193 | No |
| 75 | DSCC1 |  |  | 587 | -0.352 | -0.6230 | No |
| 76 | CACYBP |  |  | 589 | -0.352 | -0.6223 | No |
| 77 | DDX39A |  |  | 590 | -0.353 | -0.6200 | No |
| 78 | EIF4EBP1 |  |  | 592 | -0.353 | -0.6193 | No |
| 79 | CMSS1 |  |  | 593 | -0.353 | -0.6170 | No |
| 80 | HSPB11 |  |  | 594 | -0.354 | -0.6148 | No |
| 81 | GGCT |  |  | 598 | -0.356 | -0.6170 | No |
| 82 | RNASEH2B |  |  | 599 | -0.356 | -0.6147 | No |
| 83 | COMMD4 |  |  | 601 | -0.358 | -0.6139 | No |
| 84 | HIRIP3 |  |  | 603 | -0.359 | -0.6132 | No |
| 85 | ZWILCH |  |  | 604 | -0.360 | -0.6109 | No |
| 86 | PRIM2 |  |  | 607 | -0.362 | -0.6116 | No |
| 87 | ASRGL1 |  |  | 608 | -0.362 | -0.6092 | No |
| 88 | PARP2 |  |  | 609 | -0.362 | -0.6069 | No |
| 89 | WDHD1 |  |  | 610 | -0.363 | -0.6046 | No |
| 90 | POC1A |  |  | 611 | -0.363 | -0.6023 | No |
| 91 | PRTFDC1 |  |  | 613 | -0.364 | -0.6015 | No |
| 92 | BTG3 |  |  | 615 | -0.365 | -0.6007 | No |
| 93 | HNRNPF |  |  | 621 | -0.369 | -0.6058 | No |
| 94 | WDR76 |  |  | 626 | -0.373 | -0.6094 | No |
| 95 | EXOSC8 |  |  | 630 | -0.376 | -0.6115 | No |
| 96 | CCP110 |  |  | 636 | -0.381 | -0.6166 | No |
| 97 | PHF19 |  |  | 648 | -0.391 | -0.6305 | No |
| 98 | TEDC1 |  |  | 651 | -0.394 | -0.6310 | No |
| 99 | TMEM106C |  |  | 654 | -0.397 | -0.6314 | Yes |
| 100 | SHCBP1 |  |  | 655 | -0.398 | -0.6289 | Yes |
| 101 | NRGN |  |  | 657 | -0.399 | -0.6279 | Yes |
| 102 | GINS2 |  |  | 658 | -0.399 | -0.6253 | Yes |
| 103 | DCK |  |  | 659 | -0.400 | -0.6228 | Yes |
| 104 | CDT1 |  |  | 665 | -0.406 | -0.6277 | Yes |
| 105 | MNS1 |  |  | 666 | -0.408 | -0.6251 | Yes |
| 106 | SAE1 |  |  | 668 | -0.408 | -0.6240 | Yes |
| 107 | PGAM1 |  |  | 670 | -0.410 | -0.6229 | Yes |
| 108 | DSN1 |  |  | 671 | -0.410 | -0.6202 | Yes |
| 109 | NUDT1 |  |  | 674 | -0.413 | -0.6206 | Yes |
| 110 | GNG4 |  |  | 676 | -0.415 | -0.6195 | Yes |
| 111 | SAC3D1 |  |  | 679 | -0.416 | -0.6198 | Yes |
| 112 | PKMYT1 |  |  | 681 | -0.420 | -0.6186 | Yes |
| 113 | H2AFV |  |  | 684 | -0.423 | -0.6189 | Yes |
| 114 | CDCA4 |  |  | 687 | -0.424 | -0.6192 | Yes |
| 115 | RPA2 |  |  | 688 | -0.424 | -0.6165 | Yes |
| 116 | CENPQ |  |  | 689 | -0.425 | -0.6138 | Yes |
| 117 | FANCA |  |  | 691 | -0.425 | -0.6126 | Yes |
| 118 | DIAPH3 |  |  | 696 | -0.427 | -0.6159 | Yes |
| 119 | RANBP1 |  |  | 697 | -0.427 | -0.6131 | Yes |
| 120 | ANP32B |  |  | 699 | -0.431 | -0.6119 | Yes |
| 121 | CHEK1 |  |  | 704 | -0.433 | -0.6151 | Yes |
| 122 | PARPBP |  |  | 705 | -0.434 | -0.6124 | Yes |
| 123 | TIMELESS |  |  | 707 | -0.435 | -0.6111 | Yes |
| 124 | E2F1 |  |  | 708 | -0.435 | -0.6083 | Yes |
| 125 | TEX30 |  |  | 709 | -0.436 | -0.6055 | Yes |
| 126 | POLD3 |  |  | 710 | -0.437 | -0.6027 | Yes |
| 127 | LMNB2 |  |  | 713 | -0.438 | -0.6029 | Yes |
| 128 | RBBP8 |  |  | 714 | -0.440 | -0.6001 | Yes |
| 129 | RAD18 |  |  | 715 | -0.443 | -0.5973 | Yes |
| 130 | ALYREF |  |  | 716 | -0.444 | -0.5945 | Yes |
| 131 | SLC20A1 |  |  | 717 | -0.444 | -0.5917 | Yes |
| 132 | SKA1 |  |  | 719 | -0.447 | -0.5903 | Yes |
| 133 | MASTL |  |  | 721 | -0.451 | -0.5889 | Yes |
| 134 | RAD21 |  |  | 728 | -0.458 | -0.5950 | Yes |
| 135 | GINS1 |  |  | 730 | -0.459 | -0.5936 | Yes |
| 136 | BRIP1 |  |  | 734 | -0.461 | -0.5951 | Yes |
| 137 | CSE1L |  |  | 736 | -0.461 | -0.5937 | Yes |
| 138 | C19orf48 |  |  | 738 | -0.462 | -0.5922 | Yes |
| 139 | E2F8 |  |  | 741 | -0.467 | -0.5922 | Yes |
| 140 | MGME1 |  |  | 742 | -0.468 | -0.5893 | Yes |
| 141 | SMC3 |  |  | 743 | -0.470 | -0.5863 | Yes |
| 142 | TK1 |  |  | 747 | -0.480 | -0.5877 | Yes |
| 143 | MIS18A |  |  | 750 | -0.483 | -0.5876 | Yes |
| 144 | LIG1 |  |  | 752 | -0.485 | -0.5860 | Yes |
| 145 | CDCA3 |  |  | 759 | -0.497 | -0.5918 | Yes |
| 146 | TRIP13 |  |  | 760 | -0.498 | -0.5887 | Yes |
| 147 | MMS22L |  |  | 761 | -0.500 | -0.5855 | Yes |
| 148 | MXD3 |  |  | 763 | -0.502 | -0.5838 | Yes |
| 149 | GINS4 |  |  | 765 | -0.510 | -0.5820 | Yes |
| 150 | GMNN |  |  | 766 | -0.510 | -0.5788 | Yes |
| 151 | RTKN2 |  |  | 767 | -0.511 | -0.5755 | Yes |
| 152 | LRR1 |  |  | 768 | -0.512 | -0.5723 | Yes |
| 153 | CMC2 |  |  | 771 | -0.518 | -0.5719 | Yes |
| 154 | CDC45 |  |  | 773 | -0.521 | -0.5701 | Yes |
| 155 | DTYMK |  |  | 774 | -0.522 | -0.5668 | Yes |
| 156 | RFC4 |  |  | 777 | -0.527 | -0.5664 | Yes |
| 157 | DEPDC1B |  |  | 779 | -0.533 | -0.5645 | Yes |
| 158 | SKA3 |  |  | 783 | -0.539 | -0.5656 | Yes |
| 159 | CEP152 |  |  | 785 | -0.542 | -0.5636 | Yes |
| 160 | FANCD2 |  |  | 787 | -0.544 | -0.5617 | Yes |
| 161 | RPA3 |  |  | 788 | -0.550 | -0.5582 | Yes |
| 162 | NCAPD3 |  |  | 790 | -0.559 | -0.5561 | Yes |
| 163 | ATAD5 |  |  | 791 | -0.559 | -0.5525 | Yes |
| 164 | HAUS8 |  |  | 792 | -0.560 | -0.5490 | Yes |
| 165 | RACGAP1 |  |  | 796 | -0.566 | -0.5499 | Yes |
| 166 | ASF1B |  |  | 798 | -0.568 | -0.5477 | Yes |
| 167 | CENPJ |  |  | 799 | -0.568 | -0.5441 | Yes |
| 168 | RFC3 |  |  | 801 | -0.569 | -0.5420 | Yes |
| 169 | TUBB |  |  | 802 | -0.570 | -0.5384 | Yes |
| 170 | TUBB4B |  |  | 804 | -0.573 | -0.5362 | Yes |
| 171 | CENPN |  |  | 805 | -0.574 | -0.5326 | Yes |
| 172 | USP1 |  |  | 806 | -0.575 | -0.5289 | Yes |
| 173 | CDC6 |  |  | 807 | -0.584 | -0.5252 | Yes |
| 174 | CHAF1A |  |  | 810 | -0.588 | -0.5244 | Yes |
| 175 | PSMC3IP |  |  | 811 | -0.588 | -0.5207 | Yes |
| 176 | DEK |  |  | 812 | -0.591 | -0.5169 | Yes |
| 177 | FOXM1 |  |  | 814 | -0.593 | -0.5146 | Yes |
| 178 | DEPDC1 |  |  | 815 | -0.596 | -0.5108 | Yes |
| 179 | CDCA2 |  |  | 816 | -0.597 | -0.5070 | Yes |
| 180 | MCM7 |  |  | 817 | -0.599 | -0.5032 | Yes |
| 181 | LMNB1 |  |  | 818 | -0.606 | -0.4994 | Yes |
| 182 | MCM8 |  |  | 819 | -0.607 | -0.4955 | Yes |
| 183 | MND1 |  |  | 821 | -0.615 | -0.4931 | Yes |
| 184 | CKAP2L |  |  | 826 | -0.625 | -0.4951 | Yes |
| 185 | CCDC34 |  |  | 827 | -0.626 | -0.4911 | Yes |
| 186 | DNAJC9 |  |  | 828 | -0.633 | -0.4871 | Yes |
| 187 | AURKB |  |  | 829 | -0.634 | -0.4830 | Yes |
| 188 | HELLS |  |  | 830 | -0.635 | -0.4790 | Yes |
| 189 | DTL |  |  | 831 | -0.637 | -0.4749 | Yes |
| 190 | PCNA |  |  | 832 | -0.640 | -0.4709 | Yes |
| 191 | PIMREG |  |  | 833 | -0.641 | -0.4668 | Yes |
| 192 | BUB3 |  |  | 834 | -0.641 | -0.4627 | Yes |
| 193 | CENPW |  |  | 835 | -0.643 | -0.4586 | Yes |
| 194 | CDKN3 |  |  | 836 | -0.653 | -0.4544 | Yes |
| 195 | ITGB3BP |  |  | 838 | -0.661 | -0.4517 | Yes |
| 196 | ANP32E |  |  | 841 | -0.676 | -0.4504 | Yes |
| 197 | KIF22 |  |  | 842 | -0.678 | -0.4461 | Yes |
| 198 | BIRC5 |  |  | 843 | -0.679 | -0.4418 | Yes |
| 199 | CENPM |  |  | 844 | -0.682 | -0.4374 | Yes |
| 200 | NCAPH |  |  | 845 | -0.685 | -0.4331 | Yes |
| 201 | BRCA1 |  |  | 846 | -0.691 | -0.4287 | Yes |
| 202 | ZWINT |  |  | 847 | -0.692 | -0.4243 | Yes |
| 203 | SPC24 |  |  | 848 | -0.694 | -0.4199 | Yes |
| 204 | CENPH |  |  | 849 | -0.694 | -0.4154 | Yes |
| 205 | BRCA2 |  |  | 850 | -0.694 | -0.4110 | Yes |
| 206 | DHFR |  |  | 851 | -0.701 | -0.4065 | Yes |
| 207 | VRK1 |  |  | 852 | -0.711 | -0.4020 | Yes |
| 208 | CIP2A |  |  | 853 | -0.712 | -0.3975 | Yes |
| 209 | KIF2C |  |  | 855 | -0.721 | -0.3944 | Yes |
| 210 | NUF2 |  |  | 856 | -0.721 | -0.3898 | Yes |
| 211 | CENPK |  |  | 857 | -0.722 | -0.3852 | Yes |
| 212 | FAM111A |  |  | 858 | -0.727 | -0.3806 | Yes |
| 213 | KIF4A |  |  | 861 | -0.736 | -0.3789 | Yes |
| 214 | FEN1 |  |  | 862 | -0.736 | -0.3742 | Yes |
| 215 | FBXO5 |  |  | 864 | -0.742 | -0.3710 | Yes |
| 216 | RRM1 |  |  | 866 | -0.749 | -0.3677 | Yes |
| 217 | ARHGAP11A |  |  | 867 | -0.756 | -0.3629 | Yes |
| 218 | NDC80 |  |  | 869 | -0.762 | -0.3595 | Yes |
| 219 | ORC6 |  |  | 870 | -0.766 | -0.3546 | Yes |
| 220 | BUB1B |  |  | 871 | -0.766 | -0.3498 | Yes |
| 221 | CCNA2 |  |  | 872 | -0.770 | -0.3449 | Yes |
| 222 | KIF23 |  |  | 873 | -0.772 | -0.3399 | Yes |
| 223 | MELK |  |  | 874 | -0.776 | -0.3350 | Yes |
| 224 | TMPO |  |  | 875 | -0.778 | -0.3300 | Yes |
| 225 | CKAP2 |  |  | 878 | -0.781 | -0.3281 | Yes |
| 226 | NCAPG2 |  |  | 879 | -0.781 | -0.3231 | Yes |
| 227 | MCM10 |  |  | 881 | -0.798 | -0.3195 | Yes |
| 228 | H2AFX |  |  | 882 | -0.799 | -0.3144 | Yes |
| 229 | KIFC1 |  |  | 883 | -0.807 | -0.3093 | Yes |
| 230 | EZH2 |  |  | 886 | -0.810 | -0.3071 | Yes |
| 231 | FAM111B |  |  | 888 | -0.822 | -0.3034 | Yes |
| 232 | SGO1 |  |  | 889 | -0.822 | -0.2981 | Yes |
| 233 | KIF15 |  |  | 890 | -0.834 | -0.2928 | Yes |
| 234 | CDCA5 |  |  | 892 | -0.841 | -0.2890 | Yes |
| 235 | H2AFZ |  |  | 893 | -0.849 | -0.2836 | Yes |
| 236 | RAD51AP1 |  |  | 894 | -0.853 | -0.2781 | Yes |
| 237 | KPNA2 |  |  | 895 | -0.858 | -0.2727 | Yes |
| 238 | DBF4 |  |  | 896 | -0.864 | -0.2672 | Yes |
| 239 | SMC2 |  |  | 897 | -0.867 | -0.2616 | Yes |
| 240 | SGO2 |  |  | 898 | -0.869 | -0.2561 | Yes |
| 241 | PBK |  |  | 900 | -0.880 | -0.2520 | Yes |
| 242 | FANCI |  |  | 901 | -0.882 | -0.2464 | Yes |
| 243 | SMC4 |  |  | 902 | -0.886 | -0.2408 | Yes |
| 244 | DUT |  |  | 903 | -0.887 | -0.2351 | Yes |
| 245 | MIS18BP1 |  |  | 904 | -0.889 | -0.2294 | Yes |
| 246 | C21orf58 |  |  | 905 | -0.893 | -0.2238 | Yes |
| 247 | CCNE2 |  |  | 907 | -0.895 | -0.2196 | Yes |
| 248 | XRCC2 |  |  | 908 | -0.907 | -0.2138 | Yes |
| 249 | CKS1B |  |  | 909 | -0.912 | -0.2080 | Yes |
| 250 | HJURP |  |  | 910 | -0.921 | -0.2021 | Yes |
| 251 | CKS2 |  |  | 911 | -0.922 | -0.1962 | Yes |
| 252 | GTSE1 |  |  | 912 | -0.941 | -0.1902 | Yes |
| 253 | MYBL2 |  |  | 914 | -0.959 | -0.1856 | Yes |
| 254 | KNL1 |  |  | 915 | -0.963 | -0.1795 | Yes |
| 255 | CLSPN |  |  | 916 | -0.964 | -0.1734 | Yes |
| 256 | TYMS |  |  | 917 | -0.965 | -0.1672 | Yes |
| 257 | CENPU |  |  | 918 | -0.971 | -0.1610 | Yes |
| 258 | PRC1 |  |  | 919 | -0.980 | -0.1548 | Yes |
| 259 | MAD2L1 |  |  | 921 | -0.990 | -0.1500 | Yes |
| 260 | UBE2T |  |  | 922 | -0.994 | -0.1437 | Yes |
| 261 | SPC25 |  |  | 924 | -1.033 | -0.1386 | Yes |
| 262 | HIST1H1E |  |  | 925 | -1.049 | -0.1319 | Yes |
| 263 | ESCO2 |  |  | 926 | -1.067 | -0.1251 | Yes |
| 264 | UBE2C |  |  | 928 | -1.108 | -0.1195 | Yes |
| 265 | NUSAP1 |  |  | 929 | -1.112 | -0.1125 | Yes |
| 266 | TPX2 |  |  | 930 | -1.124 | -0.1053 | Yes |
| 267 | NCAPG |  |  | 931 | -1.142 | -0.0980 | Yes |
| 268 | ASPM |  |  | 932 | -1.146 | -0.0907 | Yes |
| 269 | ATAD2 |  |  | 933 | -1.162 | -0.0833 | Yes |
| 270 | CENPF |  |  | 935 | -1.200 | -0.0772 | Yes |
| 271 | HMGB2 |  |  | 937 | -1.238 | -0.0708 | Yes |
| 272 | CDK1 |  |  | 938 | -1.248 | -0.0629 | Yes |
| 273 | RRM2 |  |  | 940 | -1.403 | -0.0554 | Yes |
| 274 | MKI67 |  |  | 941 | -1.407 | -0.0465 | Yes |
| 275 | TOP2A |  |  | 942 | -1.417 | -0.0375 | Yes |
| 276 | PCLAF |  |  | 943 | -1.554 | -0.0276 | Yes |
| 277 | HIST1H1D |  |  | 944 | -1.810 | -0.0160 | Yes |
| 278 | HIST1H4C |  |  | 946 | -2.752 | -0.0000 | Yes |
Table: GSEA details [plain text format]

  

Fig 2: DOROSZKO\_U3180MG\_CLUSTER\_3: Random ES distribution      
 Gene set null distribution of ES for **DOROSZKO\_U3180MG\_CLUSTER\_3**
